# The Lifelong Impact of Bullying Behaviours on Crime Through David Farrington's Legacy

**DOI:** 10.1002/cbm.2380

**Published:** 2025-03-19

**Authors:** Louise Arseneault

**Affiliations:** ^1^ King's College London The Institute of Psychiatry, Psychology and Neuroscience London UK

I have spent a significant part of my career with a team of talented young researchers investigating how childhood bullying impacts people's mental health and overall functioning. Together over the years, we have demonstrated that the impact of childhood bullying victimisation is environmentally mediated (Arseneault et al. 2008), that children who experience bullying benefit from supportive family environments (Bowes et al. 2010) and that bullying contributes to early psychotic symptoms (Arseneault et al. 2011). We also explored the biological effects of bullying victimisation, including HPA axis dysregulation (Ouellet‐Morin et al. 2011) and inflammation (Danese et al. 2011). Our findings highlight the long‐term impact of being bullied in childhood on both mental and physical health in adulthood (Takizawa et al. 2014) and document the burden bullying places on the NHS and UK mental health services (Evans‐Lacko et al. 2017).

Whenever I presented these findings at conferences or scientific meetings, without fail someone in the audience would ask, ‘What about the children who bully others?’ My initial thought was always the same: these are children with conduct problems, and decades of research have already produced extensive knowledge about them. What more remains to be uncovered? Eventually, I decided to investigate whether children who bully others were simply children with conduct problems or if there was more to their behaviour. In doing so, I followed in the footsteps of David Farrington, who has long studied bullying behaviours and their developmental impact.

David Farrington extensively examined bullying from a criminological perspective, investigating the long‐term trajectory of children who bully others. In doing so, he drew insights from his own Integrated Cognitive Antisocial Potential (ICAP) theory, in which he aimed to explain criminal behaviour by distinguishing antisocial potential (AP) and cognition (Farrington 2020). His theory stipulates that individuals with high long‐term antisocial potential are more likely to engage in persistent offending, whereas those with low long‐term antisocial potential may only commit crimes in certain situations. It integrates psychological and social influences to demonstrate why some people offend more frequently or persistently than others. From this view, Farrington hypothesised that both bullying and violent offending are symptoms of the same underlying issues, suggesting that the later outcomes of children who bully would be similar to those involved in violent offences. His analyses invariably used data from the Cambridge Study in Delinquent Development, a prospective longitudinal cohort study of 411 men from South London, first assessed in the early 1960s. He led that study for several years, following on from his colleague, British psychiatrist Donald J. West.

With Maria Ttofi as his partner in crime, Farrington demonstrated that bullying behaviour in childhood predicts poor life outcomes, including violent behaviour and criminal convictions (Ttofi et al. 2011), drug use (Ttofi et al. 2016) and low‐status employment, even after accounting for other childhood risk factors such as antisocial and aggressive behaviours (Farrington and Ttofi 2011). Their findings align with those from 15 other prospective longitudinal studies, as reported in a meta‐analysis, which found that children who bully are approximately two‐thirds more likely to engage in violence later in life, even when controlling for other risk factors (Ttofi et al. 2012).

Overall, Farrington's work highlighted that children who engage in bullying struggle with a range of difficulties during their youth that eventually cascade into unsuccessful lives in adulthood. He further advocated for anti‐bullying programs not only as a means to improve the lives of both bullies and their victims, but also as a strategy to reduce crime and violence in later years (Farrington and Ttofi 2011). Following in Farrington's footsteps, but with a developmental approach and a mental health focus, we investigated bullying behaviours in a contemporaneous prospective longitudinal study of children born in the United Kingdom between 1994 and 1995. Our research demonstrated that bullying behaviours often co‐occur with other conduct problems in childhood (Ganesan et al. 2021), and although their developmental trajectories are distinct, they remain interconnected (Thériault‐Couture et al. 2025).

We further found that bullying behaviours and conduct problems share many of the same risk factors, including low socioeconomic status, child maltreatment, domestic violence and parental antisocial behaviour (Ganesan et al. 2021). Both bullying and conduct problems were also linked to similar adverse outcomes when children turned ages 12 and 18, such as antisocial behaviour, criminal convictions, drug use and academic difficulties, as well as symptoms of depression and social isolation. Although these findings may align with theoretical models suggesting that antisocial behaviours remain stable across the life course, they challenge the common perception that children who bully others are popular among their peers and hold higher social status.

Our findings, along with those of Farrington, support the ‘failure’ model (Patterson and Stoolmiller 1991), which posits that youth with behavioural problems often develop emotional difficulties over time due to negative experiences such as academic failure and poor relationships with family and peers (Wertz et al. 2015). Our finding that bullying behaviour predicts poor outcomes over and above antisocial behaviour echoes Farrington's early conclusions that bullying behaviours and conduct problems are closely related, but they both independently contribute to poor outcomes later in life.

Together, these findings underscore the importance of implementing and supporting anti‐bullying programs and policies. A meta‐analysis by Gaffney et al. (2019) found that school‐based anti‐bullying programs reduced bullying behaviours by approximately 20% and bullying victimisation by 15%. Additionally, such programs have shown marginal reductions in emotional problems like anxiety and depression (Guzman‐Holst et al. 2022). Whether these results should be viewed with optimism or frustration remains to be determined. What is clear, however, is that much work remains to be done to address bullying both to improve the lives of those who bully and those who are their victims.

Interventions should be embedded within broader inclusion and safety agendas, rather than implemented as standalone initiatives, and should integrate existing resources wherever possible. Crucially, interventions targeting bullying behaviours and conduct problems could benefit from greater integration, as advocated by Farrington who strongly believed in greater integration instead of the compartmentalisation of interventions based on segregated outcomes (Loeber and Farrington 2000). Programs aimed at reducing conduct problems often take a family‐based approach, focusing on parenting skills (de Graaf et al. 2008; Scott et al. 2014; Fonagy et al. 2018). These programs aim to enhance parents' knowledge, skills and confidence in managing children's behaviour, benefiting young people who bully others. These programs could not only provide potential avenues for reducing bullying behaviours but also offer much‐needed support to an already overstretched education workforce.

Although I consistently used a developmental approach to examine bullying and its impact on people's lives, my conclusions surprisingly align with those of a criminologist: much like general crime in society, bullying behaviours in schools will never be fully eradicated. Although educational institutions bear the responsibility of minimising bullying and creating a safe environment for all students, we must also equip young people with the skills and tools to avoid becoming targets of bullying, much like we do in the case of crime. For instance, people are advised to lock their doors when leaving the house or to travel in groups late at night when it is dark. These strategies aim to reduce the risk of becoming victims of assaults or robberies. Similarly, we should ensure that all young people are taught the personal and social skills necessary to prevent becoming victims of bullying. The combination of strategies aimed at preventing bullying behaviour and providing support to victims could be most effective in preventing lifelong difficulties resulting from bullying behaviour and victimisation.

However, Farrington's research on bullying behaviours was not my first contact with his work. Our paths crossed when I first moved to London in the late 1990s. I joined the *Cambridge Study in Delinquent Development* team on several occasions to learn more about the functioning of this incredible study. More recently, when we began creating discoverability platforms to increase the uptake of existing longitudinal data and facilitate mental health research, I was thrilled to see the *Cambridge Study in Delinquent Development* on our *Catalogue of Mental Health Measures* (2025) and rediscover the richness of this study. Few studies are as far‐reaching and enduring. However, what impressed me most, back then and still today, was Farrington's unwavering dedication to this study and its participants. His respect for each and every man who took part in its regular assessments, coupled with his diligent use of the data to generate new knowledge, truly stood out. I will miss his regular emails listing all his publications in the past month.

David Farrington's pioneering work on bullying and longitudinal research has left an indelible mark on the field of criminology and far beyond. His compassion and integrity as a human being will continue to inspire those who, like me, will continue to follow in his footsteps.

## Conflicts of Interest

The author declares no conflicts of interest.

## Data Availability

The author has nothing to report.
